# Thinking toward improved treatments of systemic lupus erythematosus

**DOI:** 10.1186/ar3985

**Published:** 2012-09-27

**Authors:** BH Hahn, J Grossman, B Skaggs, E Lourenco, M Wong

**Affiliations:** 1 David Geffen School of Medicine, UCLA, Los Angeles, CA, USA

## 

In 2012, recommended therapies for SLE include antimalarials, glucocorticoids, azathioprine, mycophenolate mofetil (or myfortic acid), cyclophosphamide, and other immunosuppressants. Belimumab has been added recently. Most are targeted toward adaptive immune responses. We now suspect that several pathways in innate immunity are also critical to driving SLE, including dendritic cells (major source of IFNα), and monocyte/macrophages that appear central in the damage that occurs to renal tissue in lupus nephritis. Abnormalities in neutrophils may also drive IFNα production and damage to endothelial cells. Therefore, treatments that modify these innate immune cells are of great interest. Antimalarials primarily suppress antigen-presenting cells (APC), including TLR activation; clinically they suppress disease activity and damage, but not strongly. Glucocorticoids suppress DC, monocytes and lymphocytes, with reduction of trafficking of proinflammatory cells to target tissues, but they are quite toxic. Belimumab is directed primarily at prevention of B-cell maturation and has clinical benefits that are not large when added to standard therapies.

Among the potential new therapies that influence APC is Laquinimod, a quinoline derivative administered orally, which has recently been shown to reduce the number of new MRI lesions and disability in patients with multiple sclerosis. A recent study in murine EAE shows that Laquinimod suppresses activity of DC, and prevents monocyte/macrophages from accessing the CNS. The suppressed APC functions result in reduced number of effector T cells (Th1, Th17) in target tissue. In addition, Laquinimod induces regulatory T cells and myeloid regulatory CD16^+^LyC6^+ ^cells that on adoptive transfer suppress clinical disease. We have recent data, submitted for the 2012 ACR meeting, showing similar immune alterations in a murine model of lupus nephritis, including dramatic benefits on protection of young mice from clinical disease, and regression of disease in mice started on treatment after developing heavy proteinuria. Renal damage is minimal.

Downregulation of innate immune cells to minimize their activation of adaptive immunity, and their ability to invade and initiate damage target tissues should result in not only less active acute disease but also less future damage. This approach might be discussed at the Whistler meeting.

